# Freeze-Cast Porous Textured BaTiO_3_–Polymer Composites for Energy Harvesting Applications

**DOI:** 10.1021/acsaem.5c01606

**Published:** 2025-07-22

**Authors:** Ajeet Kumar, Alex Tezcan, Zihe Li, Ruxue Yang, Florian Bouville, Guylaine Poulin-Vittrant, Hamideh Khanbareh, James Roscow, Sylvain Deville, Chris Bowen

**Affiliations:** † Department of Mechanical Engineering, 1555 University of Bath, Bath BA27AY, United Kingdom; ‡ Centre for Advanced Structural Ceramics, Department of Materials, 4615 Imperial College, London SW7 2AZ, United Kingdom; § GREMAN UMR 7347, CNRS, University of Tours, INSA−CVL, Blois 41000, France; ∥ Universite Claude Bernard Lyon 1, CNRS, Institut Lumière Matière, UMR5306, F-69100 Villeurbanne, France

**Keywords:** lead-free, porous, piezoelectric, composites, mechanical properties, energy harvesting

## Abstract

Porous piezoelectric ceramics exhibit a unique combination of high piezoelectric charge coefficients (*d*_*ij*_) and low permittivity compared to their dense counterparts, which is desirable for achieving high piezosensing and energy harvesting performance. A further enhancement in performance can be achieved by inducing crystallographic texturing within the porous lead-free piezoceramic matrix while maintaining the aligned porous structure. Here, we report a process demonstrating the use of directional freeze-casting of BaTiO_3_ platelets to fabricate lead-free porous textured BaTiO_3_ ceramics with highly aligned porosity. A high degree of alignment of the piezoelectric BaTiO_3_ platelets in the freezing direction was confirmed by using scanning electron microscopy. The degree of texturing was quantified by X-ray diffraction, yielding a Lotgering factor (LF) of ∼0.37. To enhance the mechanical strength and strain to failure for sensing and harvesting applications, the porous textured BaTiO_3_ ceramics (∼60 vol % porosity, sintered at 1150 °C for 4 h) were infiltrated with polymers (epoxy and polydimethylsiloxane) of contrasting elastic properties. The BaTiO_3_–epoxy composite structure demonstrated a strain (%) to failure of 0.93 ± 0.005 at a high failure stress of 71.6 ± 3.05 MPa, with Young’s modulus of 7.6 ± 0.02 GPa. In contrast, the BaTiO_3_–PDMS composite had a flexible nature and exhibited a lower Young’s modulus of 0.015 ± 0.0012 GPa and a higher strain (%) to failure (>22 ± 1.5). The dielectric properties, polarization–electric field loops, and piezoelectric properties were examined in detail, and the poled BaTiO_3_–epoxy composite was used to fabricate a cantilever structure to demonstrate its energy harvesting and sensing performance. This work has shown that directional freeze-casting can produce an aligned porous and textured ferroelectric microstructure for sensing or energy harvesting applications.

## Graphical abstract

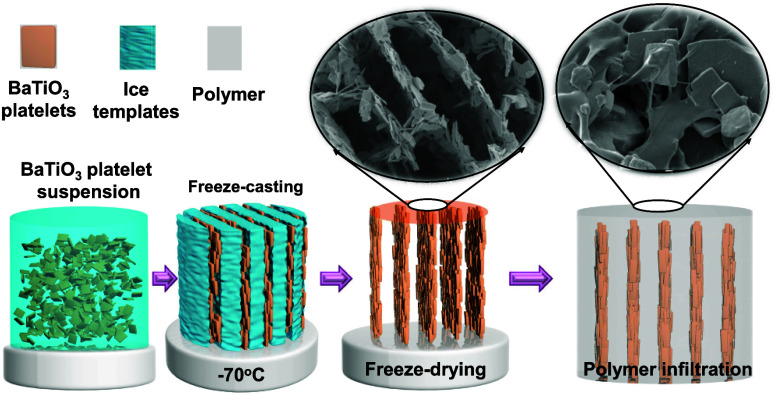

## 1 Introduction

Ferroelectrics are a group of highly polar materials that can generate electrical charge in response to mechanical stress through the direct piezoelectric effect, which makes them ideal for applications such as pressure sensors, SONAR, medical transducers, and vibration energy harvesters.^1–8^ Ferroelectric (FE) single crystals exhibit superior properties, which are attributed to the enhanced piezoelectricity from the large intrinsic (lattice) contribution compared to the extrinsic domain wall motion observed in polycrystalline ceramic counterparts.^9–12^ However, the necessity for complex fabrication processes and the difficulty of controlling the size, shape, growth parameters, and homogeneity make single-crystal materials expensive and difficult to form in complex geometries.

As a result, polycrystalline ceramics have been fabricated with a preferred crystallographic orientation, known as a ‘texture’.^13–15^ Tape casting has been used to fabricate textured ceramics, whereby 5–10 vol % of platelets are dispersed within a fine particle matrix and deposited in multiple thin layers onto a flat surface using a doctor blade to apply shear stress to align the platelets parallel to the casting direction. The resulting sample is then heat-treated to promote the growth of these aligned templates, with the degree of platelet alignment determining the final texture quality of the formed ceramics. For instance, an exceptionally high piezoelectric charge coefficient (*d*_33_) of 755 pC/N was reported for textured dense (Ba,Ca)­(Zr,Ti)­O_3_ (BCZT) ceramics, exceeding that of nontextured counterparts.^16^ While textured ceramics possess piezoelectric coefficients that are lower than their single-crystal counterparts, they are higher than conventional polycrystalline ceramics. Despite these attractive features, most commercial piezoelectric material-based devices are currently fabricated from dense, lead-based, untextured ferroelectric ceramics^17^ due to the complexities of fabricating single-crystal materials and the relatively slow layer-by-layer approach associated with the manufacture of tape-cast textured ceramics. While ferroelectric single crystals, textured ceramics, and dense polycrystalline ceramics have different microstructures, they share some common characteristics, namely, a high permittivity (ε_33_^T^). The high permittivity of these materials limits their performance figures of merit for piezoelectric sensing (*g*_33_ *= d*_33_/ε_33_^T^) and energy harvesting (*d*_33_.*g*_33_ *= d*_33_^2^/ε_33_^T^), which results in low voltage or electrical energy generation in response to an applied mechanical stress. The high density and stiffness of the materials also lead to poor acoustic impedance matching with biological tissue and water, which is undesirable for medical ultrasound and SONAR applications.

Therefore, an effort has been made to reduce the permittivity, stiffness, and density of these materials by manufacturing porous piezoelectric ceramics and composites using a directional freeze-casting method.^18–20^ The unique combination of low dielectric permittivity and high piezoelectric coefficients of aligned porous piezoelectric ceramics provides an opportunity to design new materials with attractive properties for piezoelectric sensing (force, acceleration) and energy harvesting (vibration, thermal) properties, compared to their dense counterparts.^19–21^ While porosity may be considered to have a negative impact on a piezoelectric material, there are potential benefits. First, porosity can reduce the number of oxygen vacancies in the material^21^ and can lead to an increased piezoelectric *d*_33_ charge coefficient compared to the dense material, especially if the pores are aligned in the polarization direction to facilitate poling. Second, the introduction of porosity can reduce the permittivity of the material, which can lead to improvements in the performance figures of merit for both sensing (*g*_33_ *= d*_33_/ε_33_^T^) and energy harvesting (*d*_33_ *.g*_33_ *= d*_33_^2^/ε_33_^T^).

To further increase relevant performance figures of merit (FoM), one approach is to produce porous textured materials that can exhibit improved piezoelectric coefficients due to the presence of texture, while also exhibiting low permittivity due to the presence of porosity. Freeze-casting has been used to create porous materials with highly aligned porosity. In this process, ceramic particles of the desired material are suspended in a slurry, which is subsequently frozen, and the ceramic particles are ejected from the moving solidification front, forcing them to segregate between the ice crystals that grow preferentially along the temperature gradients. The freezing parameters, such as particle size, the viscosity of the slurry, the velocity of sedimentation, and the critical freezing-front velocity at which particle trapping occurs during solidification, must be carefully controlled.^22^ There have been limited reports to date that have explored the use of the freeze-casting technique to align platelet-shaped particles.^23,24^ Hunger et al. reported on the self-assembly of platelets within a nonferroelectric alumina composite via a freeze-casting method to improve the mechanical properties.^23,25^ These materials outperformed the formed ceramics by a factor of 1.5–4 in terms of stiffness, strength, and toughness, materials that had the same amount of porosity but did not exhibit the platelet microstructure. Bouville et al. successfully produced microporous materials (alumina and sodium–potassium niobate) with aligned, lamellar ceramic walls made up of crystallographically aligned grains using a combination of modified ice templating and templated grain growth (TGG). The piezoelectric and ferroelectric properties of the porous textured sodium potassium niobate ceramics were not reported.^24,26,27^

To date, templated grain growth (TGG) and texturing methods have produced dense monolithic ceramics and composite microstructures with improved properties compared to nontextured ceramics (e.g., mechanical, piezoelectric).^28,29^ However, these processing techniques are unable to produce a composite architecture that combines a controlled macroscopic porosity with textured ceramic layers. Therefore, we propose here a freeze-casting approach for fabricating porous, composite templated grain growth structures. We aim to texture the porous material to significantly reduce the permittivity while increasing the piezosensing and energy harvesting performance. The process is attractive due to its simplicity and potential to produce various geometries, while the directional freezing produces highly aligned porosity to facilitate the polarization of the ferroelectric materials. The key challenge is to use directional freezing to align ferroelectric seed crystal templates in a common orientation. In this study, platelets of lead-free BaTiO_3_ piezoelectric ceramics were aligned vertically in the freezing direction by the freeze-casting technique. This porous templated structure of BaTiO_3_ piezoelectric ceramics was then infiltrated with polymers of contrasting mechanical properties, namely, an epoxy and polydimethylsiloxane (PDMS), to fabricate a composite structure, as shown in Figure 1. The highly aligned BaTiO_3_–epoxy and BaTiO_3_–PDMS piezoelectric–polymer composite structures were characterized for their mechanical, dielectric, ferroelectric, and energy harvesting properties. This process of templating lead-free porous piezoelectric ceramics and fabrication of composite structures is reported for the first time and provides a route toward crystallographic texturing of porous BaTiO_3_ ceramic matrices.

**Figure 1:**
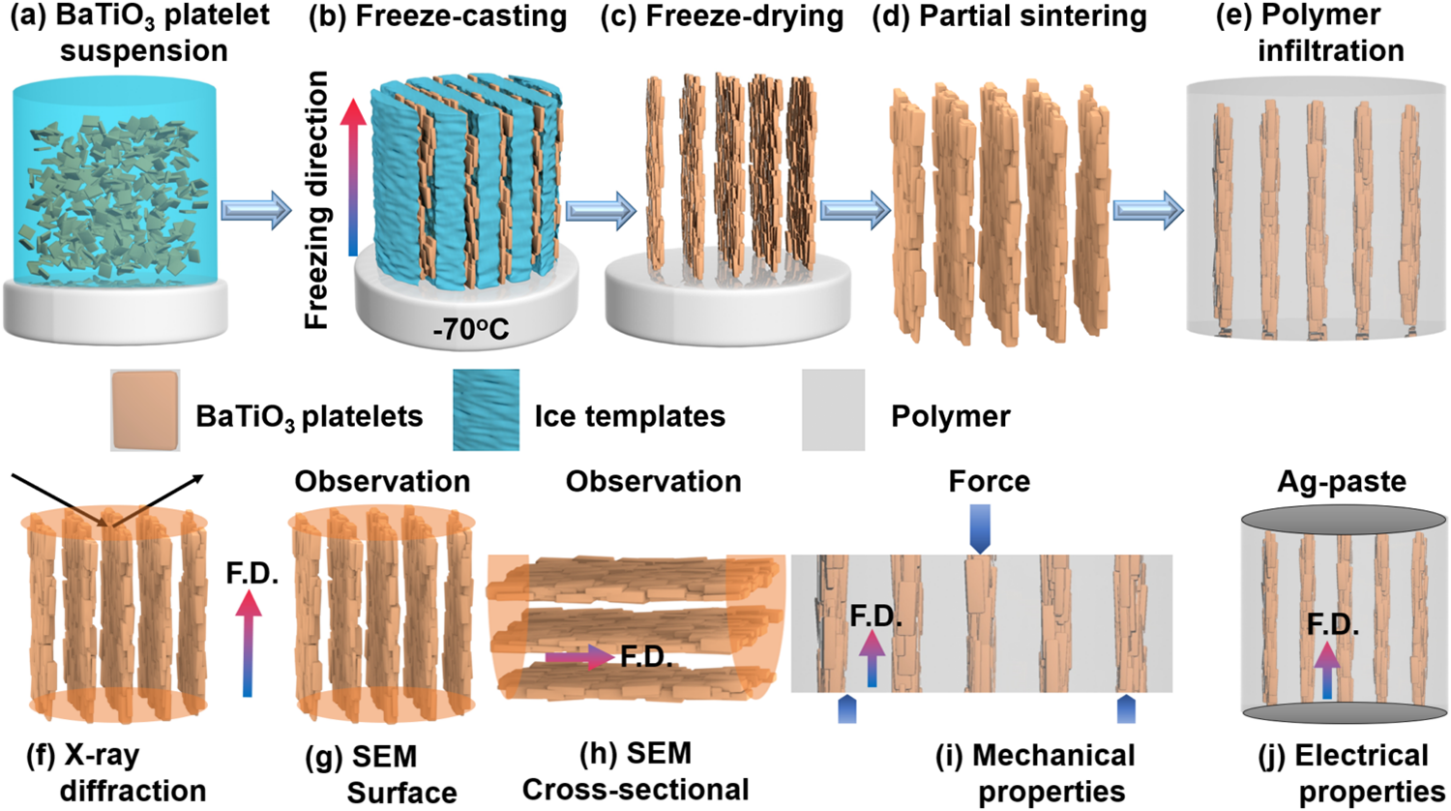
Schematic of the process to fabricate BaTiO_3_ platelet–polymer composite scaffold structure. (a) Preparation of a slurry of BaTiO_3_ platelets mixing in water, binder, and dispersant. (b) BaTiO_3_ platelets are aligned by ice templates in the freezing direction (freeze-casting). (c) Freeze-drying was used to remove these ice crystals. (d) Porous scaffold structure of BaTiO_3_ platelets is partially sintered to provide strength to the platelet assembly and to maintain their shape and size. (e) Infiltration of the porous structure with different polymers to fabricate BaTiO_3_ platelets–polymer composites. Schematic of the freezing direction (F.D.) and measurement direction of (f) X-ray diffraction, scanning electron microscopy (SEM) of the (g) top surface, (h) cross-sectional, (i) mechanical properties, and (j) electrical properties measurement of the porous textured ceramics.

## 2 Experimental Procedure

Highly aligned BaTiO_3_ templated ceramics with ∼60 vol % porosity were fabricated using commercially available BaTiO_3_ platelets (Entekno Ind. Tech. & Nano Mat. Corp., Turkey). The platelet size distribution in terms of length, width, thickness, and aspect ratio is shown in Figure S1. The freeze-casting method was used to achieve the desired porosity, whereby the BaTiO_3_ platelets were mixed with distilled water, 1 wt % of binder (poly­(vinyl alcohol), Sigma-Aldrich, molar mass = 86.09 g/mol, density = 1.19 g/cm^3^), and a dispersant (acrylic acid, Sigma-Aldrich, molar mass = 72.06 g/mol, density = 1.05 g/cm^3^) to form a slurry. The slurry was mixed for 2 h using ball milling (Capco test equipment, UK) to produce a homogeneous mix and then poured into a mold (diameter = 1.3 cm, height = 2.3 cm), which was kept on the top of a metal cooling plate (20 cm × 20 cm) that was already cooled to −70 °C using an ultralow temperature circulator (RP2090, Lauda Scientific, Germany). After freezing, a freeze-dryer (Mini LyoTrap, LTE Scientific Limited, UK) was used to remove the ice crystals from the porous aligned BaTiO_3_ platelets. The freeze-dried samples were then partially sintered at a temperature of 1150 °C for 4 h (Elite Thermal Systems Limited, UK) to produce a small degree of sintering between the platelets to produce porous scaffold structures that were sufficiently robust for polymer impregnation. A schematic of the freeze-casting and freeze-drying process is shown in Figure 1(a)–(e). At first, BaTiO_3_ platelets were mixed with distilled water to form a slurry (Figure 1(a)), which was freeze-cast (Figure 1(b)) and freeze-dried (Figure 1(c)) to fabricate the textured BaTiO_3_ ceramics with highly aligned porosity (∼60 vol %). This structure consists of vertical ceramic and porous columns along the freezing direction. This structure was partially sintered at a temperature of 1150 °C for 4 h to produce a small degree of sintering between the platelets to produce porous scaffold structures that were sufficiently robust for polymer impregnation (Figure 1(d)). This porous structure was then infiltrated with either an epoxy or a PDMS polymer, which filled the aligned vertical pores (Figure 1(e)). The epoxy and PDMS both had the same volume fraction in the textured BaTiO_3_ ceramics, since this was determined by the pore fraction in the porous textured ceramic. The sintered porous BaTiO_3_ textured ceramics were sectioned by using a diamond wire saw (STX 202A, MTI Corporation).

The freezing direction (F.D.) and measurement direction of each characterization are shown in Figure 1(f)–(j). In this work, studies on phase analysis and mechanical and electrical properties are carried out parallel to the freezing direction. X-ray diffraction (XRD, STOE STADI P, STOE & Cie GmbH, Germany) patterns and scanning electron microscopy (SU3900, Hitachi, Japan) images were taken to investigate the crystal and microstructures of the partially sintered porous BaTiO_3_ textured ceramics. To measure the electrical properties and fabricate an energy harvester, the partially sintered porous and textured BaTiO_3_ ceramics were infiltrated with epoxy (EPO-TEK 301, Epoxy Technology, Inc) and polydimethylsiloxane (PDMS, SYLGARD 184 Silicone Elastomer, The Dow Chemical Company, UK) elastomer to enhance their mechanical strength and strain to failure. To impregnate the porous BaTiO_3_ ceramic, samples were immersed in a mold filled with epoxy and PDMS (in vacuum) and cured at room temperature for 48 h. The BaTiO_3_–epoxy and BaTiO_3_–PDMS composites were then sectioned, and the electrodes were painted on opposite sides using silver paste. The frequency-dependent dielectric properties of unpoled BaTiO_3_–epoxy and BaTiO_3_–PDMS composites were measured using a precision impedance analyzer (4294A, Agilent Technologies). The ferroelectric polarization vs electric field (*P–E*) hysteresis loops of the porous BaTiO_3_–epoxy and BaTiO_3_–PDMS composites were measured using a Radiant hysteresis loop tracer (Precision Premier II, Radiant Technologies). The composite samples were poled using the corona poling technique, where a high DC voltage of 14 kV (Glassman High Voltage, Inc., UK) was applied from a point source at ∼60 °C for 60 min to pole the composite samples (the distance between the sample and the point source was ∼2.5 cm). The applied DC voltage of 14 kV provided an optimum balance between maximizing the corona poling voltage and preventing dielectric breakdown of the material or composite. The piezoelectric charge coefficient (*d*_33_) of the poled composites was measured by using a piezometer (Piezotest, Piezometer, UK). To measure the Young’s modulus of the composites, a mechanical three-point bend test was performed using samples with dimensions (length x width x thickness) of 40 × 4 × 4 mm^3^ (model 3369, Instron, Zwick-Roell, UK). A poled BaTiO_3_–epoxy composite (1 × 0.5 × 0.1 cm^3^) was mounted onto an aluminum cantilever (10 × 0.5 × 0.1 cm^3^) attached to a shaker (Bruel & Kjaer VTS Ltd., UK) that provided a mechanical excitation to evaluate the sensing and energy harvesting performance. A function generator was used to provide a signal to the voltage amplifier. The output voltage of the voltage amplifier was then used as an input signal for the shaker. The generated voltage was measured as a function of load resistance and used to charge a storage capacitor.

## 3 Results and Discussion

### 3.1 Phase and Microstructure Analysis of Porous Scaffold of BaTiO_3_ Platelets

Figure 2(a) presents the XRD pattern for freeze-cast textured BaTiO_3_ porous ceramics. Generally, for the polycrystalline BaTiO_3_ ceramics, the peak intensities of all (hkl) planes are dictated by the crystal structure of the ceramics.^30^ Both (001)-oriented single crystals and textured ceramics exhibit higher intensities of (001), (002), etc. planes compared to the other planes. In this study, an increase in the intensity of the (001), (100), (002), and (200) peaks is also observed, suggesting that a degree of texturing of the BaTiO_3_ ceramic is occurring in the (001) direction compared with the nontextured sample. For textured piezoceramics, the degree of texture along the (001) orientation was reported in terms of the Lotgering factor (LF). For samples textured along the (001) direction, the corresponding LF can be determined using eq 1, based on the XRD intensity of both random and textured ceramics^31–33^

1 $$\mathrm{LF}=\frac{p-p_{0}}{1-p_{0}}$$

where *p* is calculated from the bulk material being tested and *p*_0_ is for a powdered material. The parameter *p* denotes the fraction of the summation of the peak intensities corresponding to the preferred orientation axis compared to the summation of all diffraction peaks in particle-oriented materials. The parameter *p*_0_ is calculated from randomly powdered materials. As a result, LF = 0 corresponds to a random orientation, while LF = 1 corresponds to a single crystallographic orientation.

**Figure 2:**
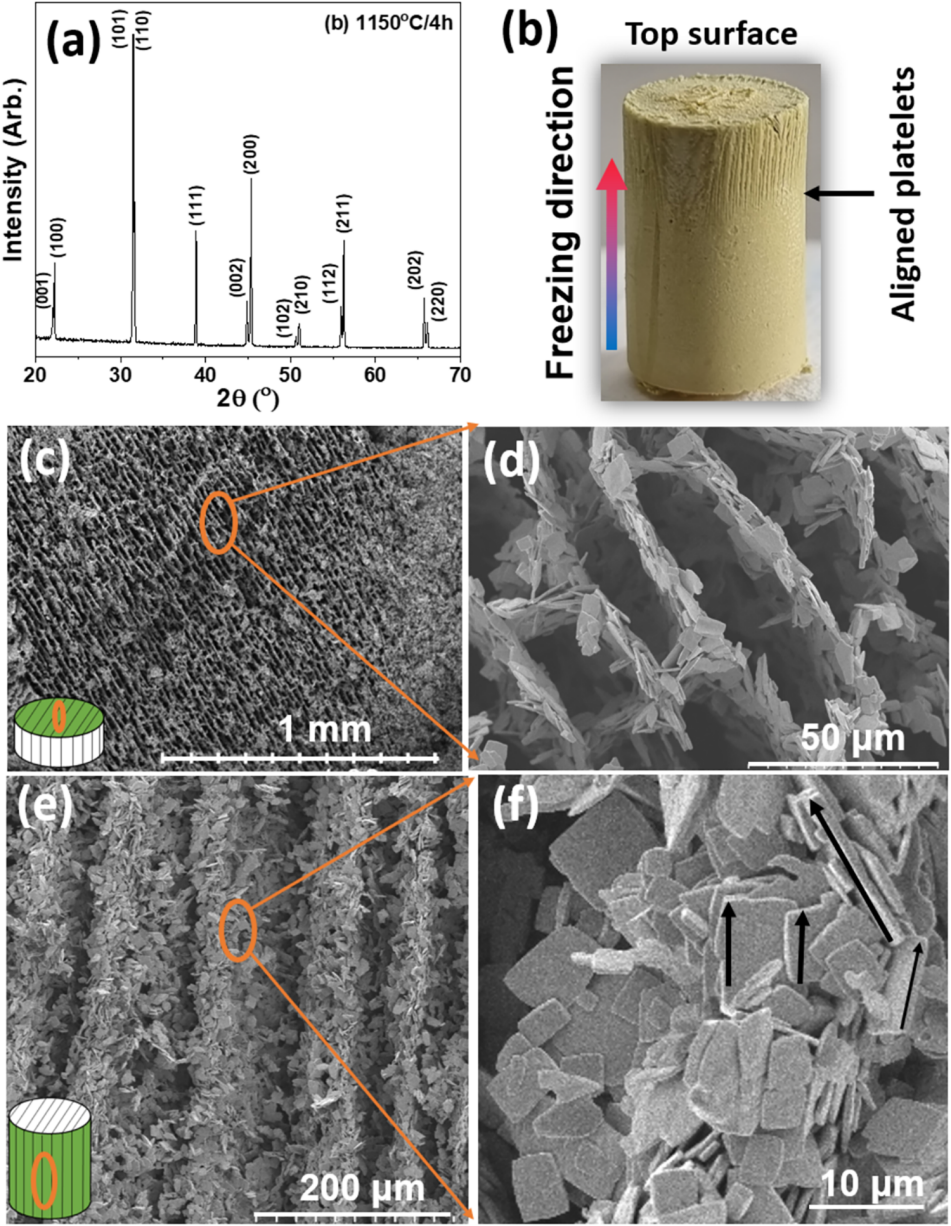
(a) X-ray diffraction pattern and (b) picture of the textured BaTiO_3_ porous sintered ceramics (diameter = 1.3 cm, height = 2.3 cm). (c), (d) Top surface and (e), (f) cross-sectional scanning electron microscopy images of porous scaffold structure of BaTiO_3_ platelets, partially sintered at 1150 °C for 4 h. The arrow in (f) represents the alignment of platelets in the freezing direction.

The LF for the porous BaTiO_3_ textured ceramics was 0.37, which is lower than samples made by tape casting^34^ but represents a good degree of alignment for the porous ceramic structure, higher than that reported on freezing under a flow of lead-free sodium potassium niobate (LF = 0.21).^24^ These results are highly encouraging in terms of both texture and aligned porosity. Figure 2(b) shows the picture of textured BaTiO3 porous sintered ceramics. Figure 2(c),(d) shows the scanning electron microscopy (SEM) images of the upper surface of directionally solidified and textured BaTiO_3_ porous ceramics (1150 °C/4 h) and provides a visual confirmation of a high degree of alignment of the BaTiO_3_ platelets during directional freezing. The cross-sectional SEM images confirm that these BaTiO_3_ platelets are aligned throughout the ceramic volume, not only on the upper surface (Figure 2(e)). A higher magnification image of Figure 2(e) clearly shows the alignment of platelets in the freezing direction; see Figure 2(f).

### 3.2 Mechanical Properties of Textured BaTiO_3_–Polymer Composites

Two different polymers were infiltrated into the porous scaffolds to produce textured BaTiO_3_ porous ceramics with sufficient mechanical stability to fabricate a sensing or harvesting device.^35,36^ Figure S2 shows textured BaTiO_3_–epoxy and BaTiO_3_–PDMS composites with different geometries. It was found that the polymers occupied the pore channels after infiltration while also maintaining the scaffold structure and platelet alignment. Figure S3 shows a SEM image of the textured BaTiO_3_–epoxy composite, while Figure 3(a),(b) shows cross-sectional SEM images of the textured BaTiO_3_–PDMS composite. These images clearly show that the polymer not only infiltrates the columnar pore structure produced by the directional freeze-casting process but also covers the BaTiO_3_ platelets so that they are effectively surrounded by the polymer matrix, thereby providing mechanical stability composites. The regions related to the PDMS matrix and BaTiO_3_ platelets are marked in Figure 3(a),(b).

**Figure 3:**
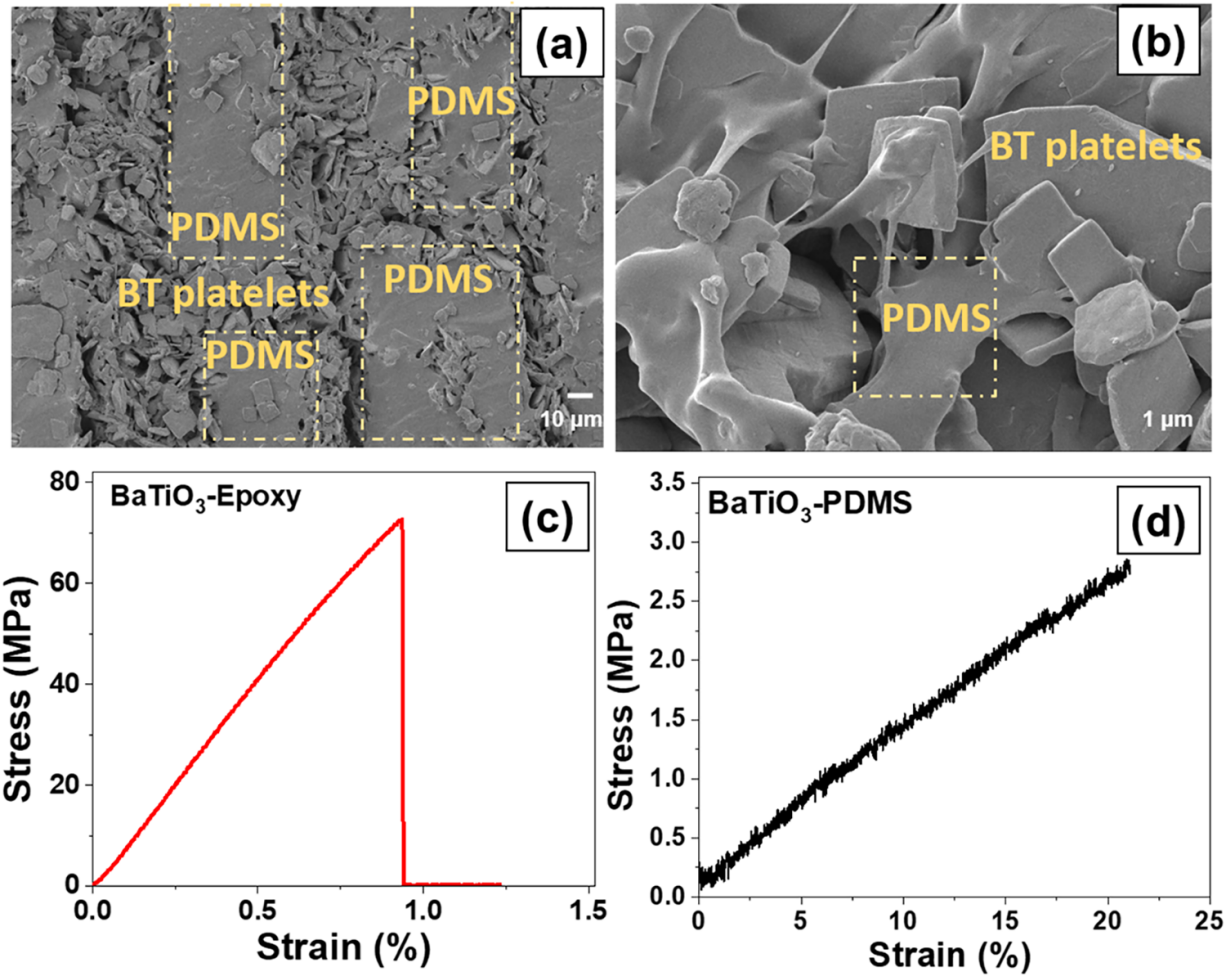
(a, b) Cross-sectional scanning electron microscopy image of the textured BaTiO_3_–PDMS composite. Yellow dotted rectangles show the PDMS-rich regions of the composites. Stress–strain curves for the textured (c) BaTiO_3_–epoxy and (d) BaTiO_3_–PDMS composites.

Three-point bend tests were conducted to determine the mechanical properties of the textured BaTiO_3_–epoxy and BaTiO_3_–PDMS composites (Figure S4). The failure stress, failure strain, and Young’s modulus for the textured BaTiO_3_–epoxy composite were found to be 71.6 ± 3.05 MPa, 0.93 ± 0.005%, and 7.6 ± 0.02 GPa, respectively. The PDMS also occupies the pores in the composite and effectively supports the composite structure, resulting in a low Young’s modulus (15 ± 1.2 MPa) along with the ability to be deformed to a high level of curvature and strain above 22 ± 1.5%. The BaTiO_3_–PDMS composite exhibits a lower Young’s modulus compared to the BaTiO_3_–epoxy composite, and this is primarily due to PDMS having a significantly lower Young’s modulus (0.56 MPa) compared to the epoxy (approximately 3.5 GPa); the greater degree of infiltration by the PDMS between the BaTiO_3_ platelets also facilitates a reduction in stiffness. The mechanical properties of the textured BaTiO_3_–epoxy and BaTiO_3_–PDMS composites are summarized in Table 1.

**Table 1:** Summary of Mechanical, Dielectric, Ferroelectric, and Piezoelectric Properties of Textured BaTiO_3_–Polymer Composites

	BaTiO_3_–epoxy	BaTiO_3_–PDMS
pore fraction (vol %)	60	60
σ_f_ (MPa)	71.6 ± 3.05	2.84 ± 0.03
*ε*_fail_ (%)	0.93 ± 0.005	22 ± 1.5
*E* (GPa)	7.6 ± 0.02	0.015 ± 0.0012
*ε*_r_ (RT, 1 kHz)	73	53
tanδ (RT, 1 kHz)	0.024	0.007
*P*_max_ (μC/cm^2^)	0.97	1.43
*P*_r_ (μC/cm^2^)	0.18	0.37
*E*_max_ (kV/cm)	87	92
*E*_c_ (kV/cm)	13.24	19.50
*d*_33_ (pC/N)	25	12
*g*_33_ (10^–3^ Vm/N)	39	26
energy harvesting FoM (*d*_33_.*g*_33_) (10^–12^ m^2^/N)	0.98	0.31

### 3.3 Dielectric, Ferroelectric, and Piezoelectric Properties of Textured BaTiO_3_–Polymer Composites

Figure 4(a),(b) shows the frequency-dependent dielectric properties of the textured BaTiO_3_–epoxy and BaTiO_3_–PDMS composite structures in the range of 1 kHz to 1 MHz. The relative permittivity (ε_r_) and dielectric loss (tan δ) of the textured BaTiO_3_–epoxy composite are ε_r_ = 73 and tan δ = 0.024, and those for the textured BaTiO_3_–PDMS are ε_r_ = 53 and tan δ = 0.007. Both the relative permittivity and the loss of the composites are lower than those of the dense BaTiO_3_ ceramic prepared using the BaTiO_3_ platelets (ε_r_ = 612, tan δ = 0.039), summarized in Table 1. The lower dielectric permittivity and loss values are a result of the formation of aligned BaTiO_3_ platelets that are suspended structures in a highly insulating and low-loss polymer matrix, where the epoxy and PDMS polymers infiltrate the BaTiO_3_ platelet scaffold structure. This can be observed in Figure 3(b) for the PDMS-based composite and in Figure S3 for the epoxy-based composite. The relative permittivity and dielectric loss of the pure epoxy are *ε*_r_ = 4 and tanδ = 0.016 and those for the pure PDMS are ε_r_ = 2.7 and tan δ = 0.002, which is responsible for the lower dielectric properties of the textured BaTiO_3_–polymer composites. The dielectric loss of the BaTiO_3_–PDMS composite is low in comparison to the BaTiO_3_–epoxy composite due to the lower dielectric loss of PDMS (tan δ = 0.002), compared to epoxy (tan δ = 0.016), along with the greater degree of infiltration by the PDMS between the BaTiO_3_ platelets, due to its lower viscosity, leading to the platelets being suspended in a highly insulating and low-loss polymer matrix.

**Figure 4:**
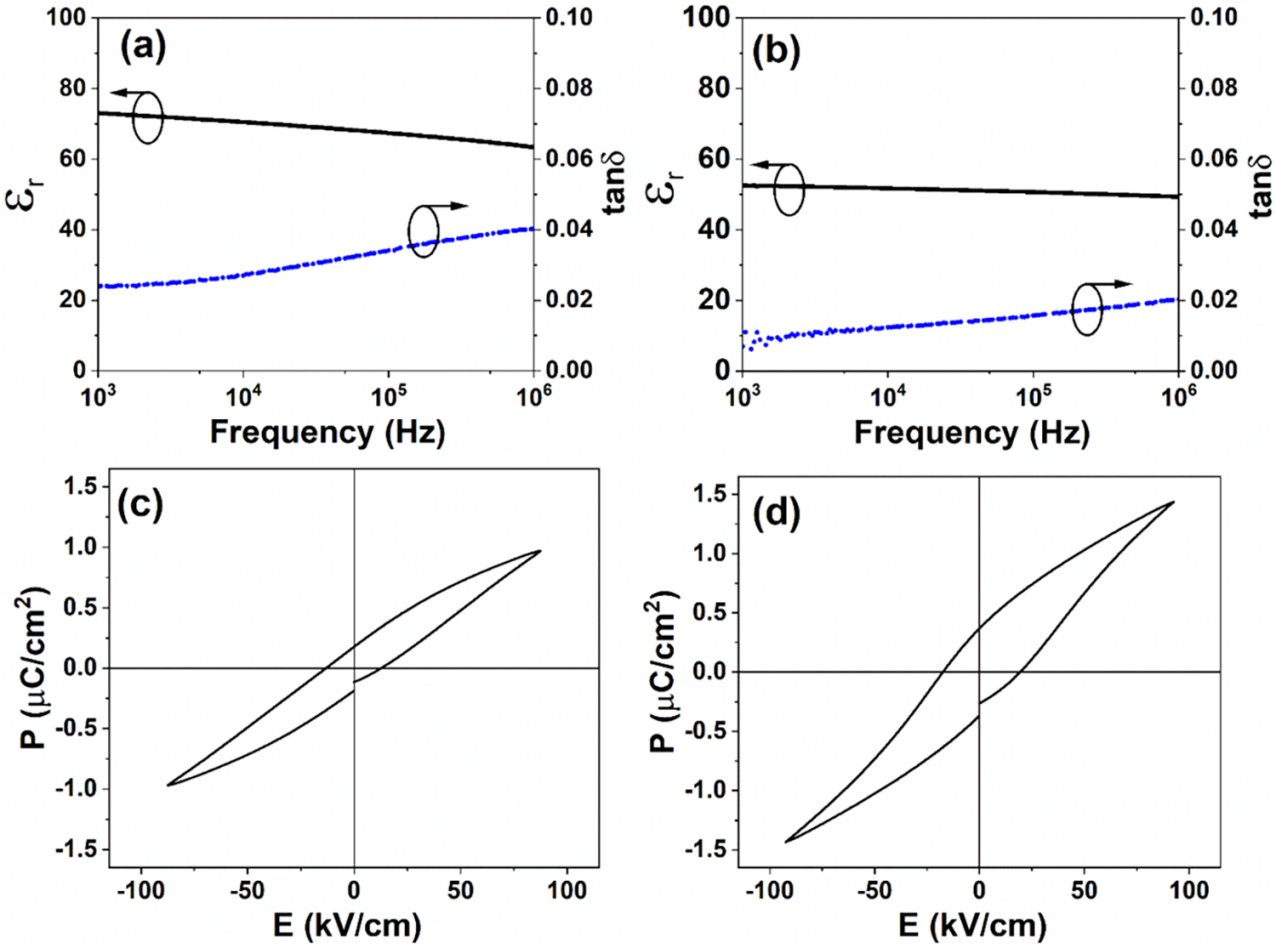
Dielectric permittivity and dielectric loss for the textured (a) BaTiO_3_–epoxy and (b) BaTiO_3_–PDMS composites. Polarization versus electric field (*P*–*E*) hysteresis loops for the textured (c) BaTiO_3_–epoxy and (d) BaTiO_3_–PDMS composites.

Polarization–electric field (*P*–*E*) ferroelectric hysteresis loops were measured for both the textured BaTiO_3_–epoxy and BaTiO_3_–PDMS composites to confirm the ferroelectric nature, as shown in Figure 4(c),(d). The textured BaTiO_3_–epoxy composite showed a maximum polarization of *P*_max_ ∼ 0.97 μC/cm^2^, remanent polarization of *P*_r_ ∼ 0.18 μC/cm^2^, and a coercive field of *E*_c_ ∼ 13.24 kV/cm. The textured BaTiO_3_–PDMS composite had a higher maximum polarization of *P*_max_ ∼ 1.43 μC/cm^2^, higher remanent polarization of *P*_r_ ∼ 0.37 μC/cm^2^, and a larger coercive field of *E*_c_ ∼ 19.50 kV/cm. The ferroelectric properties of the textured BaTiO_3_–epoxy and BaTiO_3_–PDMS composite structures are influenced by the nature of polymer infiltration and their properties. The higher *P*_max_ and *P*_r_ for the textured BaTiO_3_–PDMS composite, compared to the epoxy-based composite, is possibly related to the much lower Young’s modulus (*Y*) of the PDMS (*Y* ∼ 1.5 MPa)^37,38^ compared to the epoxy (*Y* ∼ 3.4 to 4.6 GPa),^39,40^ which reduces the degree of polymer mechanical clamping, which can reduce the degree of polarization when subjected to an electric field.^41^ The higher *E*_c_ of the textured BaTiO_3_–PDMS composite, compared to those of the epoxy-based composites, is also likely to be a result of the low-viscosity PDMS infiltrating the composites and coating the platelets to a greater extent, thereby necessitating a high applied electric field to induce switching of domains in the BaTiO_3_ platelets. The PDMS also has lower relative permittivity (*ε*_r_ = 2.7) compared to the epoxy (*ε*_r_ = 4), which can lead to a larger fraction of the applied electric field being present in the low-permittivity matrix phase.^42^

To understand the effect of polymer infiltration on the domain switching behavior of textured BaTiO_3_ ceramics, electric field and frequency-dependent *P–E* hysteresis loops, as well as current (*I–E*) loops, were also measured for textured BaTiO_3_–epoxy and BaTiO_3_–PDMS composites (Figures S5 and S6). As illustrated in Figure S5, *P–E* hysteresis loops open up with an increase in the applied electric field for both textured BaTiO_3_–epoxy and BaTiO_3_–PDMS composites. The *P–E* loops do not exhibit fully saturated square hysteresis loops owing to the presence of the polymer, since the application of a higher applied electric field leads to electrical breakdown before complete switching of the domains can be achieved. For the textured BaTiO_3_–epoxy and BaTiO_3_–PDMS composites, the *I–E* loops measured at 20 kV/cm reflect the dielectric response of the material without any clear sign of domain switching; however, peaks in the *I–E* loops, recorded at the highest applied electric field, confirm the contribution from domain switching at their coercive field. The *P–E* hysteresis loops measured at frequencies of 10 and 100 Hz for textured BaTiO_3_–epoxy and BaTiO_3_–PDMS composites do not show any significant change (Figure S6) and exhibit some opening of the loop. The existence of a ferroelectric response is also confirmed by piezoelectric *d*_33_ measurements, which are now described.

The textured BaTiO_3_–epoxy and BaTiO_3_–PDMS composites were poled by applying a DC electric field at 60 °C for 60 min. Corona poling was used, where the samples are placed in an electric field, which provides a uniform poling field. Since there is no direct electric contact between the sample and the current source, unlike direct contact poling, the potential for dielectric breakdown is reduced.^43^ While the presence of a low-permittivity polymer can reduce the ability to polarize a ferroelectric ceramic–polymer and limit the level of poling, directional freezing has been employed here to produce highly aligned and anisometric pore (and polymer) channels in the poling direction. Ferroelectric ceramics, whereby highly anisometric pores have been aligned along the poling field direction using a directional forming process, such as freeze-casting, effectively avoid the serial connection of ceramic and air phases in the direction of the poling field and lead to improved poling efficiency. The textured BaTiO_3_–epoxy and BaTiO_3_–PDMS composites show the piezoelectric charge coefficients of *d*_33_ ∼ 25 and 12 pC/N, respectively. The textured BaTiO_3_–epoxy composite exhibits a higher piezoelectric coefficient compared to the BaTiO_3_–PDMS composite, and this may be due to the higher permittivity of the epoxy, and the low viscosity of the PDMS leads to the polymer coating the platelets to a greater extent, thereby limiting the degree of polarization of the BaTiO_3_ platelets. The lower relative permittivities of the pure epoxy and PDMS are responsible for the lower dielectric, ferroelectric, and piezoelectric properties of textured BaTiO_3_–polymer composites, but as outlined above, it improves the strain to failure. All electrical properties of the textured BaTiO_3_–epoxy and BaTiO_3_–PDMS composites are summarized in Table 1, where it can be observed that the low permittivity of the composites leads to relatively good *g*_33_ voltage coefficients.^29^

### 3.4 Energy Harvesting Properties of BaTiO_3_ Platelets–Polymer Composites

The mechanical stability (Figure 3) and robustness of textured BaTiO_3_–epoxy and BaTiO_3_–PDMS composites make them suitable for fabricating a piezoelectric cantilever structure to harvest energy from mechanical vibrations. The textured BaTiO_3_–epoxy composite shows higher values of *d*_33_, *g*_33_, and energy harvesting FoM (*d*_33_ × *g*_33_) than the textured BaTiO_3_–PDMS composite used for the energy harvesting study. The schematic of the piezoelectric cantilever structure is shown in Figure 5(a), and the actual setup is shown in Figure S7. To demonstrate the energy harvesting capabilities, a textured BaTiO_3_–epoxy composite was used as a piezoelectric element (1 × 0.5 × 0.1 cm^3^) that was bonded to the aluminum cantilever (10 × 0.5 × 0.1 cm^3^) with a tip mass (∼24 g) to fabricate an energy harvester. The dimensions of the piezoelectric element were selected based on the previous study.^2^ The thickness of the piezoelectric element was kept at ∼1 mm, similar to the thickness of the cantilever beam, in order to transfer the highest strain to the material to produce the optimum voltage.^21^ The proof mass was tuned to provide the great deflection at the oscillating frequency and therefore maximum power output and maximum strain to the composite to evaluate its applicability. This textured BaTiO_3_–epoxy composite piezoelectric cantilever was attached to a shaker to provide mechanical vibrations, which were controlled by the input signal voltage and frequency. During the measurement, the signal generator generates a sine wave signal with a voltage peak-to-peak amplitude (*V*_pp_) and a frequency to drive the shaker, which was optimized by keeping the voltage constant and changing the frequency and measuring the output voltage of the energy harvester (Table S1). The shaker parameters were optimized so that the piezoelectric energy harvester produced the highest output voltage and with a peak-to-peak voltage of 15 V and a frequency of 15 Hz, as shown in Figure S8. Figure S8 shows the output voltage waveforms of the textured BaTiO_3_–epoxy energy harvester with a driving voltage of 15 V and frequency of 15 Hz while varying the proof mass of 10, 17, and 24 g. Under these periodic mechanical vibrations, the sample outputs periodic electrical signals, which are then collected by an electrometer.

**Figure 5:**
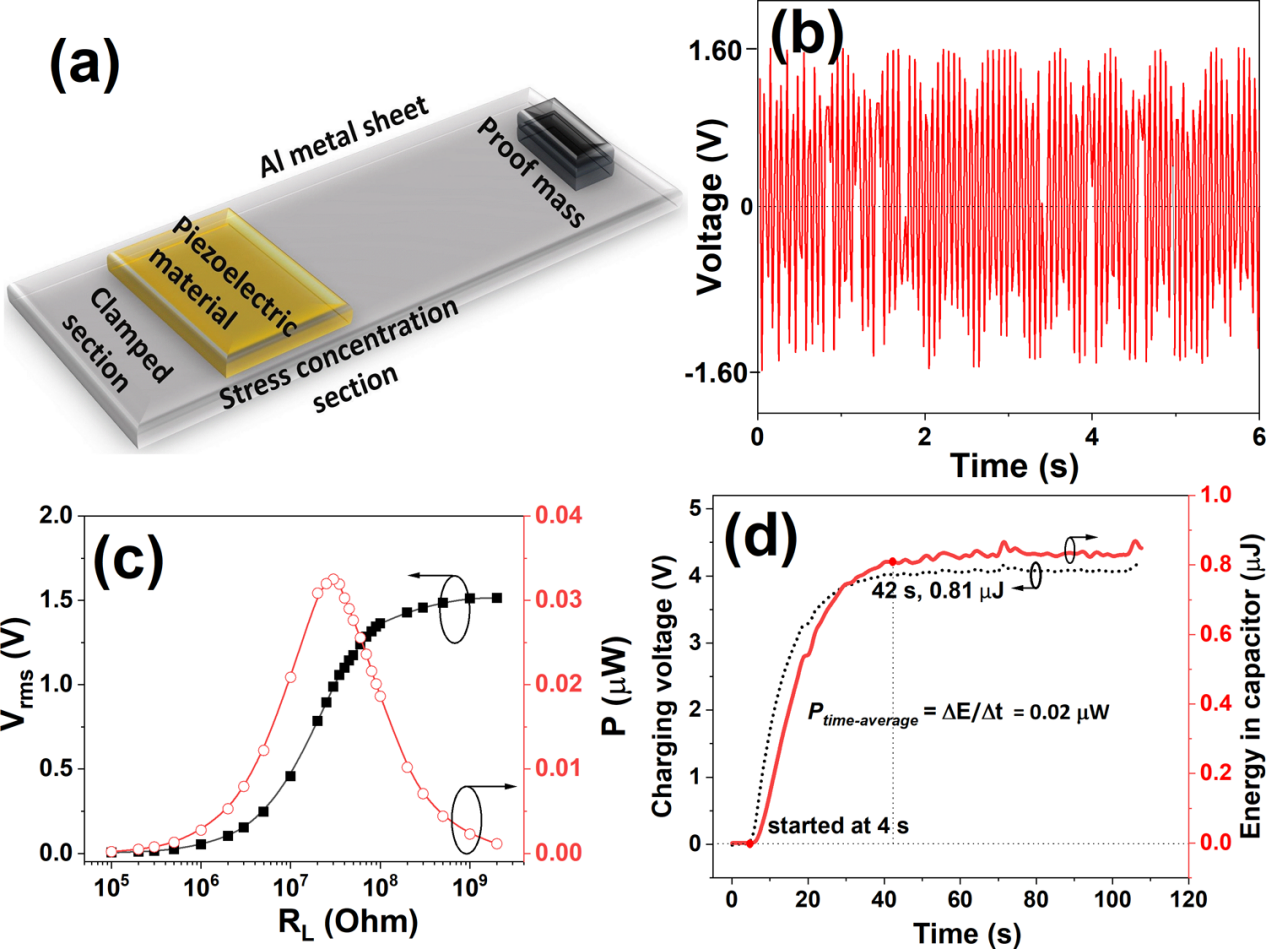
(a) Schematic of the textured BaTiO_3_–epoxy composite piezoelectric energy harvester used for this study. (b) Generated output voltage waveform from the cantilever with mechanical vibrations. (c) Output root-mean-square voltage and power as a function of load resistance. (d) Capacitor charging curve as a function of time from the textured BaTiO_3_–epoxy composite cantilever beam energy harvester by applying mechanical vibrations of 15 Hz with an amplitude of 15 V.

As shown in Figure 5(b), the textured BaTiO_3_–epoxy composite generates an output voltage of approximately 3.2 *V*pp. Figure 5(c) illustrates the output voltage and power of the textured BaTiO_3_–epoxy-based energy harvester as a function of load resistance (*R*_L_). The output voltage increases with an increase in *R*_L_ and saturates at a maximum root-mean-square (RMS) voltage (*V*_rms_) of 1.51 V. The power of the textured BaTiO_3_–epoxy-based energy harvester is calculated using the formula *P* = *V* ^2^/*R*, where *V* represents the output *V*_rms_ voltage and *R* is the load resistance (*R*_L_). At a load resistance of approximately 30 MΩ, the textured BaTiO_3_–epoxy-based energy harvester achieves the highest power of 0.033 μW. The output *V*_rms_ voltage from the textured BaTiO_3_–epoxy-based energy harvester was rectified by using a bridge rectifier to charge a capacitor, as shown in Figure 5(d). A capacitor of 10 μF is fully charged in less than 1 min (42 s).

In this study, the energy harvester is a simple device structure to demonstrate the feasibility of using these textured materials in an application such as harvesting or sensing. To demonstrate the feasibility of these materials to be applied for vibration sensing or harvesting, with potential to sustain the typical mechanical load, we have fabricated an energy harvester, which can withstand high-strain, high-frequency mechanical vibrations compared with dense or noninfiltrated porous materials. The textured BaTiO_3_–epoxy composite generates an output voltage of ∼3.2 *V*_pp_, *V*_rms_ ∼ 1.51 V, power ∼ 0.033 μW, and volume power density of 0.66 μW/cm^3^ (*R*_L_ ∼ 30 MΩ), which may not be the highest power output compared with the other energy harvesters but generates sufficient voltage to charge the capacitor. When compared with energy harvesters fabricated with single-crystal fibers or that harvest the energy from more than one excitation,^44^ the volume power density of textured BaTiO_3_–epoxy composite is sufficiently high to power the sensors and devices at remote locations. Future studies aim to improve the level of texturing of the material, poling conditions, high piezoelectric charge coefficients, and high energy harvesting figures of merit. These results show that the textured BaTiO_3_ porous ceramics can be employed to fabricate a piezoelectric energy harvester with comparable energy harvesting properties.^29,45^

## 4 Conclusions

This paper presents a method to fabricate textured porous ceramics based on BaTiO_3_ platelets by using a freeze-casting approach. The potential to align ferroelectric platelets due to the forces generated by the developing ice-freezing front during directional freezing is reported and confirmed by scanning electron microscopy images. XRD patterns of textured BaTiO_3_ porous ceramics indicate a degree of texturing of ∼0.37. The epoxy- and polydimethylsiloxane-infiltrated textured BaTiO_3_ porous ceramics show high mechanical strengths and high strain to failure. The textured BaTiO_3_–epoxy composite structure exhibits failure stress (71.6 ± 3.05 MPa), failure strain (0.93 ± 0.005%), and Young’s modulus (7.6 ± 0.02 GPa). The textured BaTiO_3_–PDMS composite structure shows a flexible structure with a higher strain (∼22 ± 1.5%). The lower value of dielectric, ferroelectric, and piezoelectric properties of textured BaTiO_3_–polymer composites is due to the low relative permittivity of the pure epoxy and PDMS. Under the periodic mechanical vibrations, the textured BaTiO_3_–epoxy composite piezoelectric cantilever generates an output voltage of ∼3.2 *V*_pp_, a *V*_rms_ of 1.51 V, and the highest power of 0.033 μW. The capacitor of 10 μF is fully charged in less than 1 min using a rectified output voltage of the energy harvester. This study provides insight into complete control over the template position and orientation in freeze-casting and will open new possibilities for synthesizing low-cost, high-performance textured materials and devices in various geometries for a range of applications.

## Supplementary Material



## Notes

XRD pattern, SEM image, and platelet size distribution of BaTiO_3_ platelets; Images of the textured BaTiO_3_–polymer composites with different geometries; Surface SEM images of textured BaTiO_3_–epoxy composite with freezing direction; Pictures of the mechanical testing setup and the textured BaTiO_3_–polymer composite samples; Electric field dependent *P*–*E* hysteresis and *I–E* loops for BaTiO_3_–polymer composite samples; Frequency-dependent *P*–*E* hysteresis loops for BaTiO_3_–polymer composite samples; Experimental setup of the textured BaTiO_3_–epoxy composite piezoelectric energy harvester; The output voltage waveforms of the textured BaTiO_3_–epoxy energy harvester with different proof masses; Table for generated output voltage of textured BaTiO_3_–epoxy energy harvester with different optimization parameters of proof mass, signal voltage, and frequency (PDF)
