# Supplementary material for: Freeze-Cast Porous Textured BaTiO3–Polymer Composites for Energy Harvesting Applications
Source: ACS Appl Energy Mater. 2025 Jul 22;8(15):11437–46. doi: 10.1021/acsaem.5c01606 (PMC12344699; doi:10.1021/acsaem.5c01606)
Supplement: Supplementary file 1 [file ae5c01606_si_001.pdf]

Supporting information (SI)

Title: Freeze-Cast Porous Textured $BaTiO_3$-Polymer Composites for Energy Harvesting Applications

Journal: ACS Applied Energy Materials

Authors: Ajeet Kumar[1,*], Alex Tezcan[1], Zihe Li[1], Ruxue Yang[2], Florian Bouville[2], Guylaine Poulin-Vittrant[3], Hamideh Khanbareh[1], James Roscow[1], and Sylvain Deville[4], and Chris Bowen[1]

[1]Department of Mechanical Engineering, University of Bath, Bath, BA2 7AY, United Kingdom

[2]Centre for Advanced Structural Ceramics, Department of Materials, Imperial College, London SW7 2AZ, United Kingdom

[3]GREMAN UMR 7347, CNRS, University of Tours, INSA–CVL, Blois, 41000, France

[4]Universite Claude Bernard Lyon 1, CNRS, Institut Lumière Matière, UMR5306, F-69100, Villeurbanne, France

*Email: ak3829@bath.ac.uk

Supplementary figures:

Fig. S1: (a) X-ray diffraction pattern and (b) Scanning electron microscopy image of $BaTiO_3$ platelets. Platelet size distribution in (c) length (d) width (e), thickness and (f) aspect ratio.

Fig. S2: (a) Images of the textured $BaTiO_3$-polymer composites with different geometries. Images of (a) square textured $BaTiO_3$-epoxy composite (b)-(c) textured $BaTiO_3$-PDMS composite samples with different geometries, and (d)-(e) top view of the textured $BaTiO_3$-PDMS composite with respective freezing directions (F.D.). Here, the grey 'dot' represents the F.D. that is perpendicular to the plane of the underlying paper.

Fig. S3: (a)-(b) surface (c)-(d) scanning electron microscopy image of textured $BaTiO_3$-epoxy composite with freezing directions (F.D.). Yellow and white rectangles show the epoxy and $BaTiO_3$ platelet-rich regions of the composites.

Fig. S4: (a) Pictures of the textured $BaTiO_3$-polymer composite samples used for mechanical testing. Mechanical test of the textured (b) $BaTiO_3$-epoxy composite and (c) $BaTiO_3$-PDMS composite. Here, the black 'dot' represents the freezing direction that is perpendicular to the plane of the underlying paper.

Fig. S5: Electric field dependent (a)-(c) polarization vs electric field (*P*-*E*) hysteresis loops and (b)-(d) domain switching current vs electric field loops for textured $BaTiO_3$-epoxy and $BaTiO_3$-PDMS composites, respectively, measured at 10Hz.

Fig. S6: Frequency-dependent polarization vs electric field (*P*-*E*) hysteresis loops for textured (a) $BaTiO_3$-epoxy and (b) $BaTiO_3$-PDMS composites, measured at their respective highest applied electric field.

Fig. S7: Experimental set-up of the textured $BaTiO_3$-epoxy composite piezoelectric energy harvester used for this study.

Fig. S8: The output voltage waveforms of the textured $BaTiO_3$-epoxy energy harvester with proof mass of (a) 10 g, (b) 17 g and (c) 24 g while driving voltage (15 V) and frequency (15 Hz) were kept constant.

Supplementary Tables:

Table S1. The change in generated output voltage of textured $BaTiO_3$-epoxy energy harvester with different optimization parameters of proof mass, signal voltage and frequency.

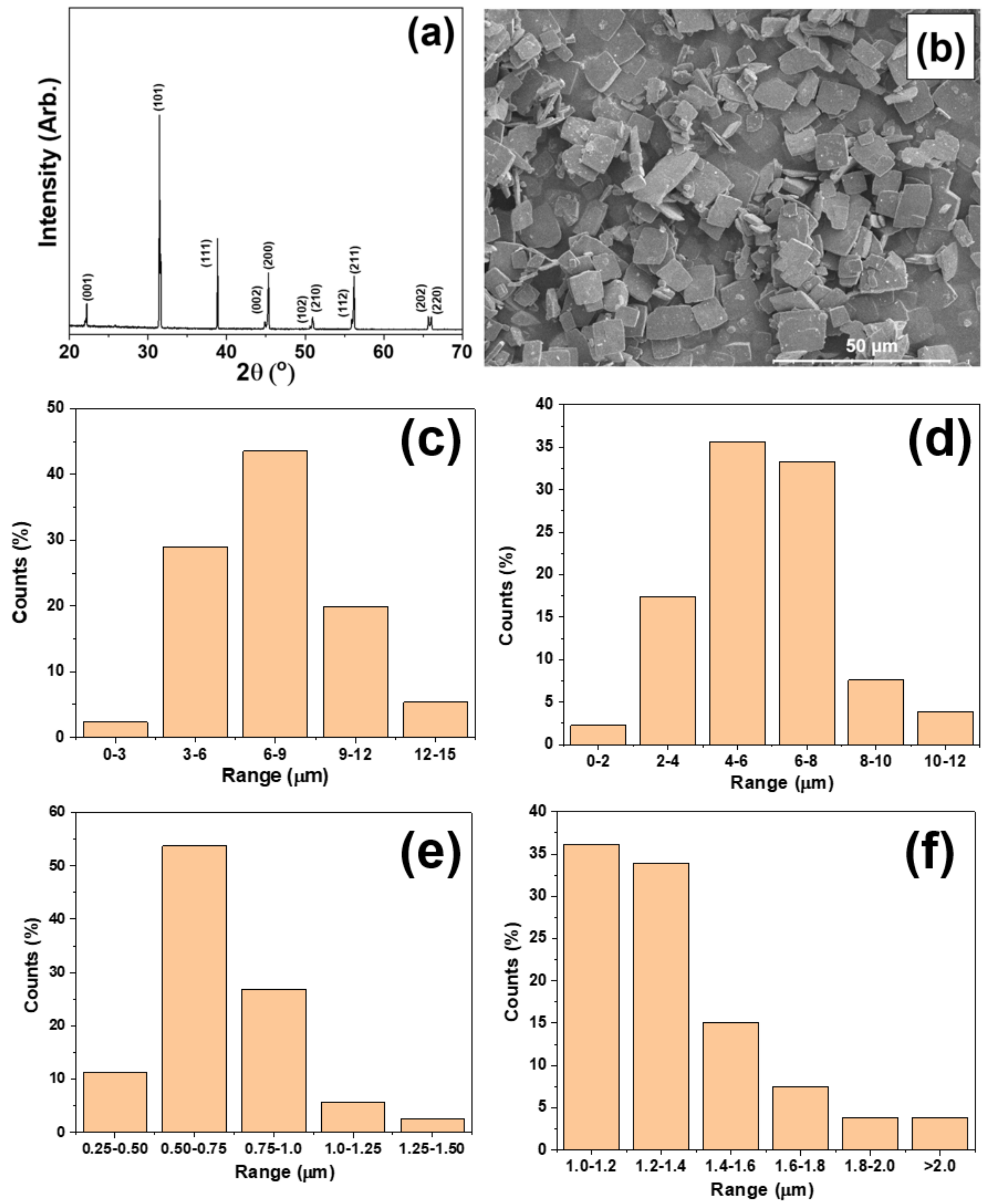

Fig. S1: (a) X-ray diffraction pattern and (b) Scanning electron microscopy image of $BaTiO_3$ platelets. Platelet size distribution in (c) length (d) width (e), thickness and (f) aspect ratio.

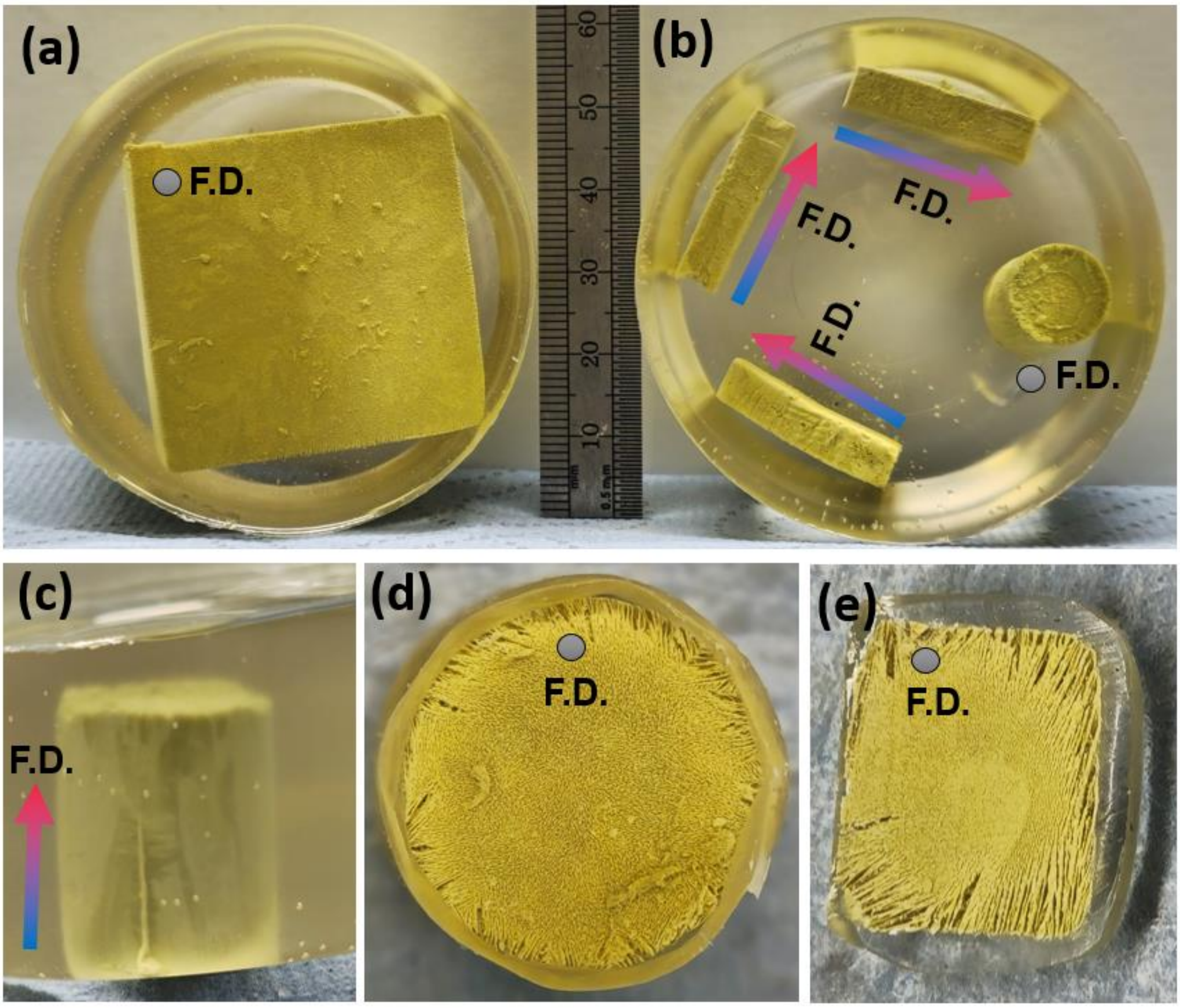

Fig. S2: (a) Images of the textured $BaTiO_3$-polymer composites with different geometries. Images of (a) square textured $BaTiO_3$-epoxy composite (b)-(c) textured $BaTiO_3$-PDMS composite samples with different geometries, and (d)-(e) top view of the textured $BaTiO_3$-PDMS composite with respective freezing directions (F.D.). Here, the grey 'dot' represents the F.D. that is perpendicular to the plane of the underlying paper.

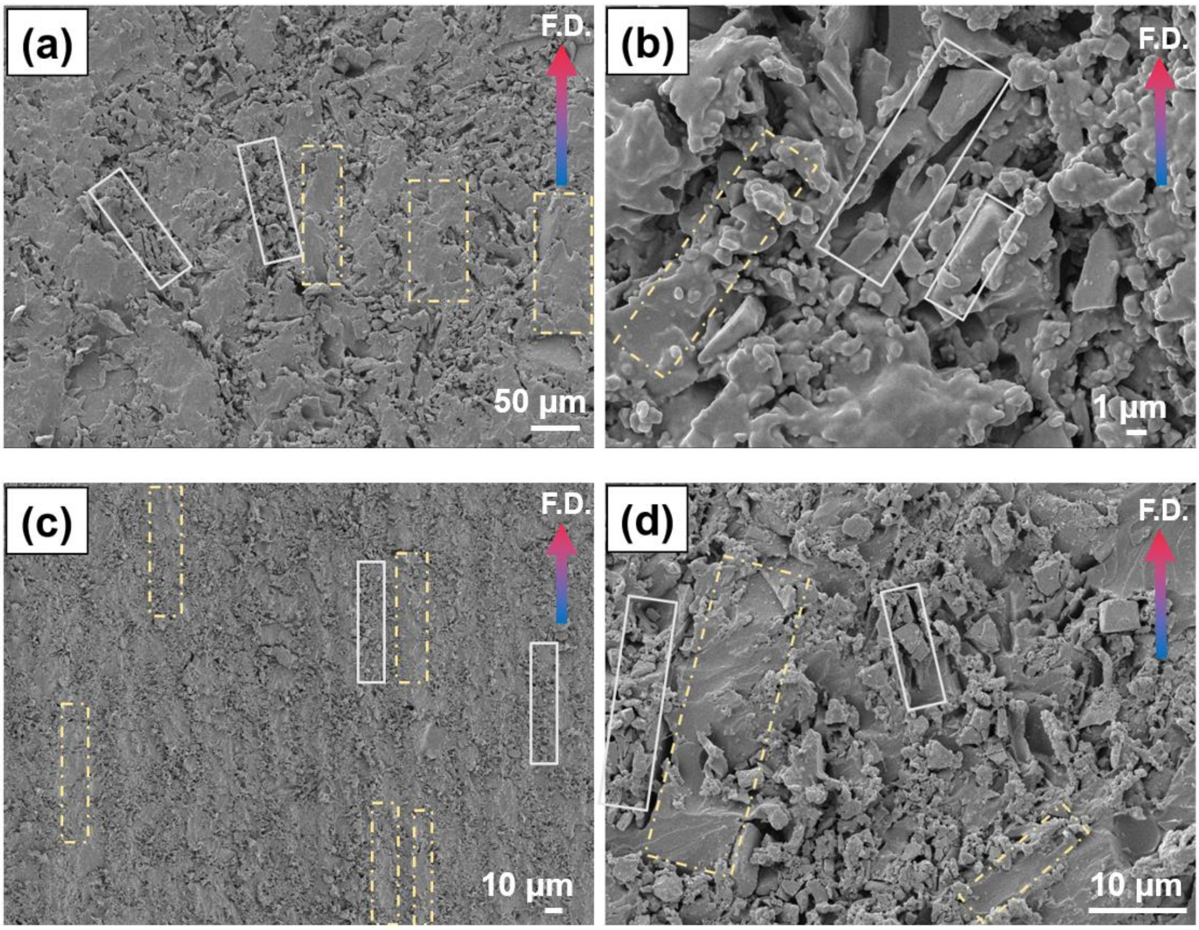

Fig. S3: (a)-(b) surface (c)-(d) scanning electron microscopy image of textured $BaTiO_3$-epoxy composite with freezing directions (F.D.). Yellow and white rectangles show the epoxy and $BaTiO_3$ platelet-rich regions of the composites.

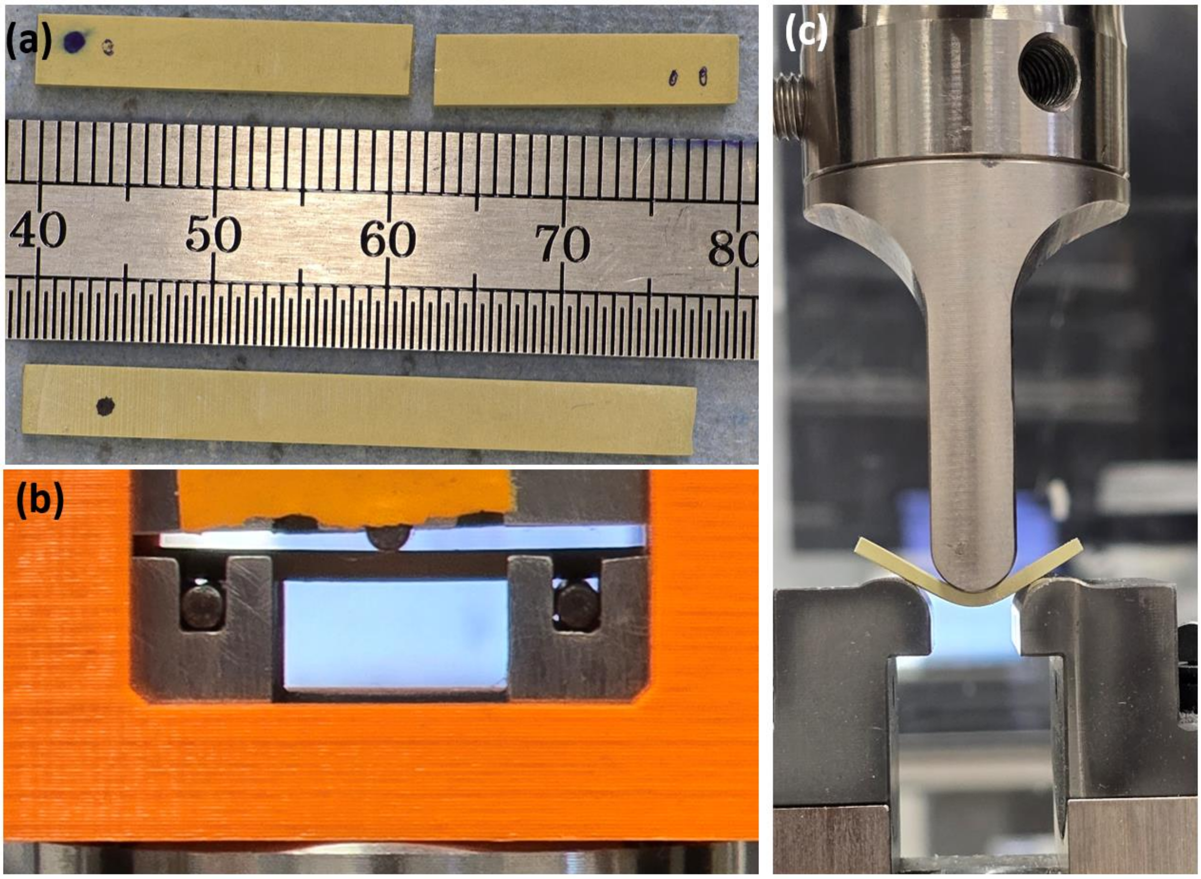

Fig. S4: (a) Pictures of the textured $BaTiO_3$-polymer composite samples used for mechanical testing. Mechanical test of the textured (b) $BaTiO_3$-epoxy composite and (c) $BaTiO_3$-PDMS composite. Here, the black 'dot' represents the freezing direction that is perpendicular to the plane of the underlying paper.

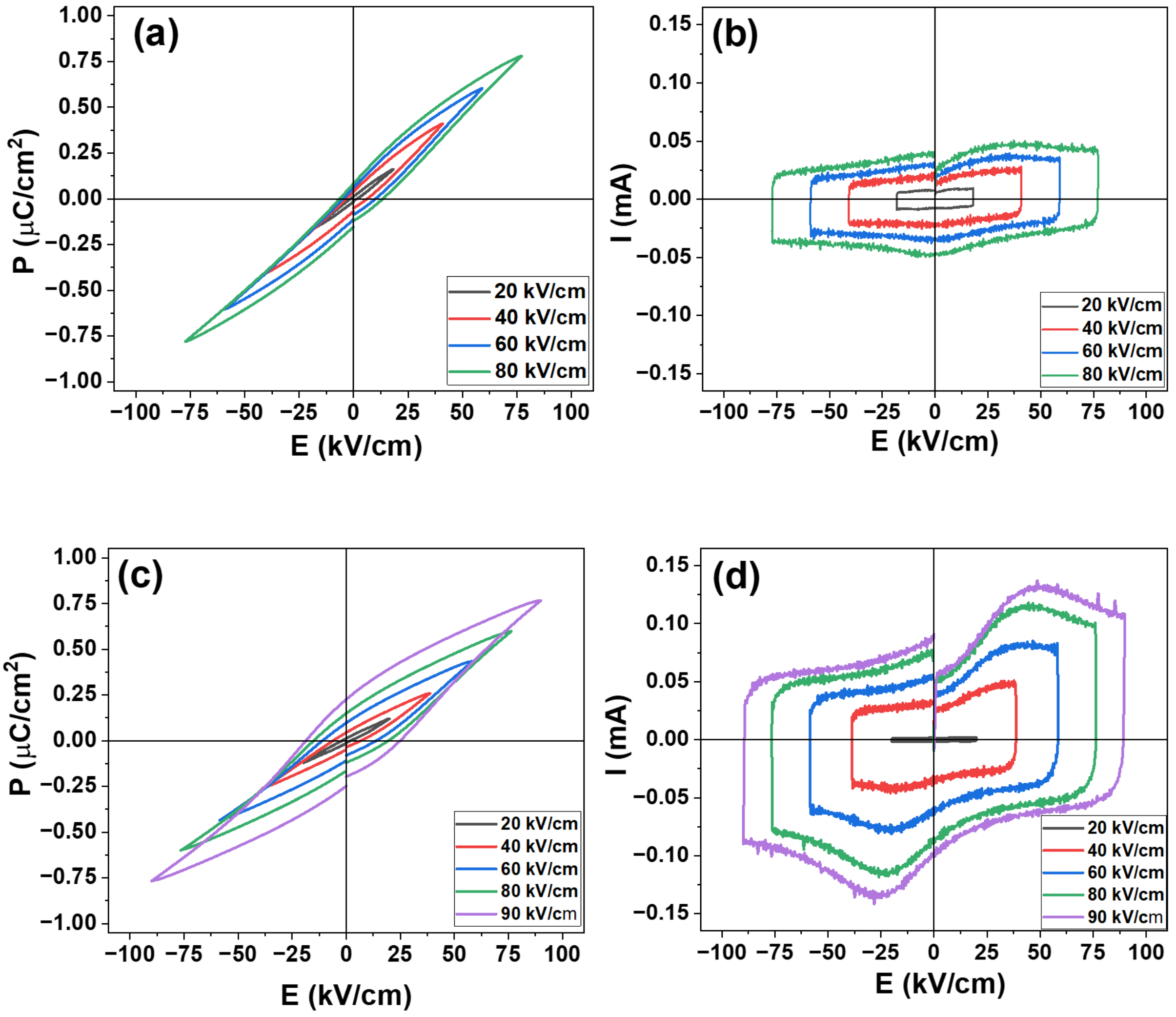

Fig. S5: Electric field dependent (a)-(c) polarization vs electric field (*P*-*E*) hysteresis loops and (b)-(d) domain switching current vs electric field loops for textured $BaTiO_3$-epoxy and $BaTiO_3$-PDMS composites, respectively, measured at 10Hz.

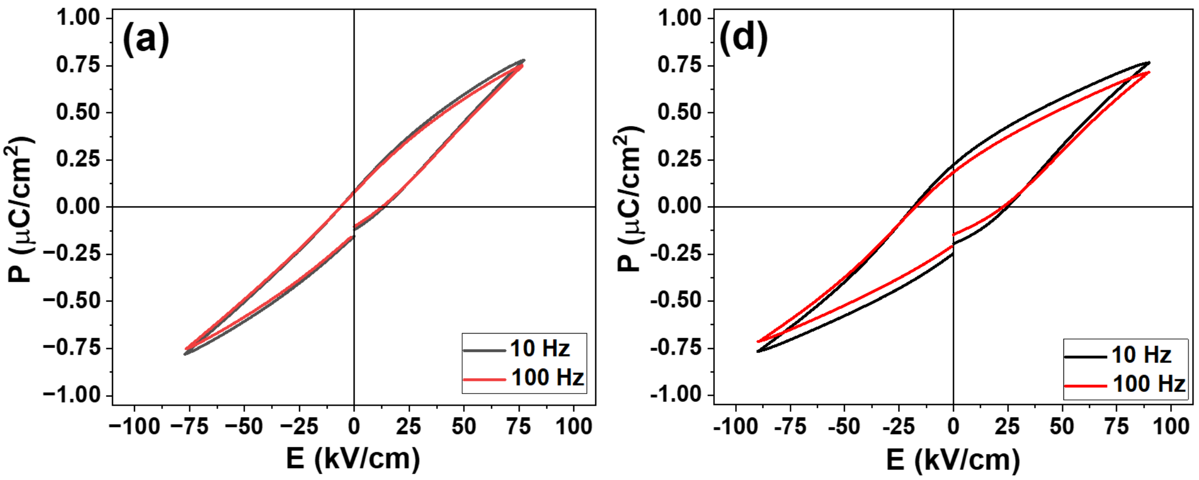

Fig. S6: Frequency-dependent polarization vs electric field (*P*-*E*) hysteresis loops for textured (a) $BaTiO_3$-epoxy and (b) $BaTiO_3$-PDMS composites, measured at their respective highest applied electric field.

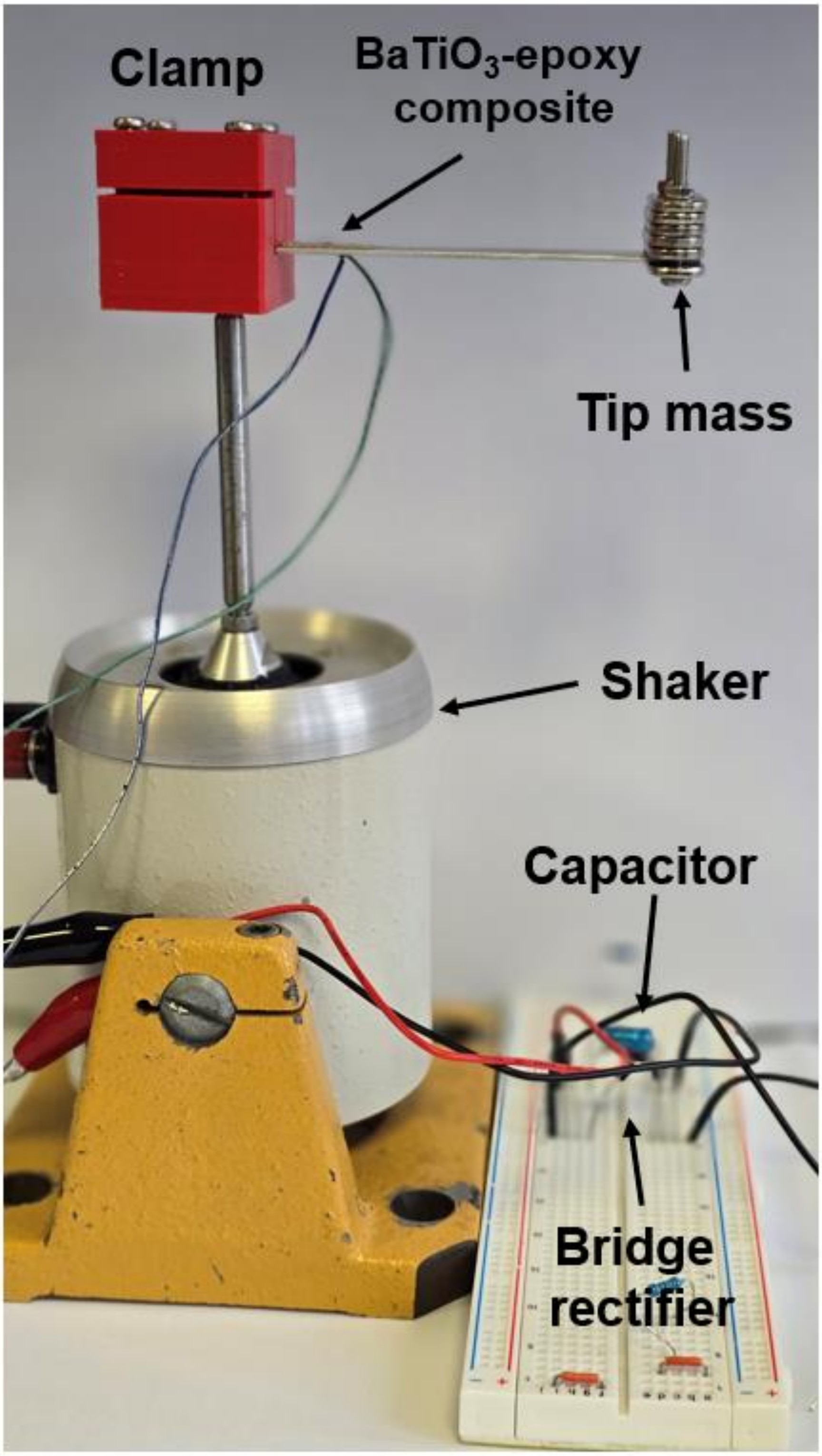

Fig. S7: Experimental set-up of the textured $BaTiO_3$-epoxy composite piezoelectric energy harvester used for this study.

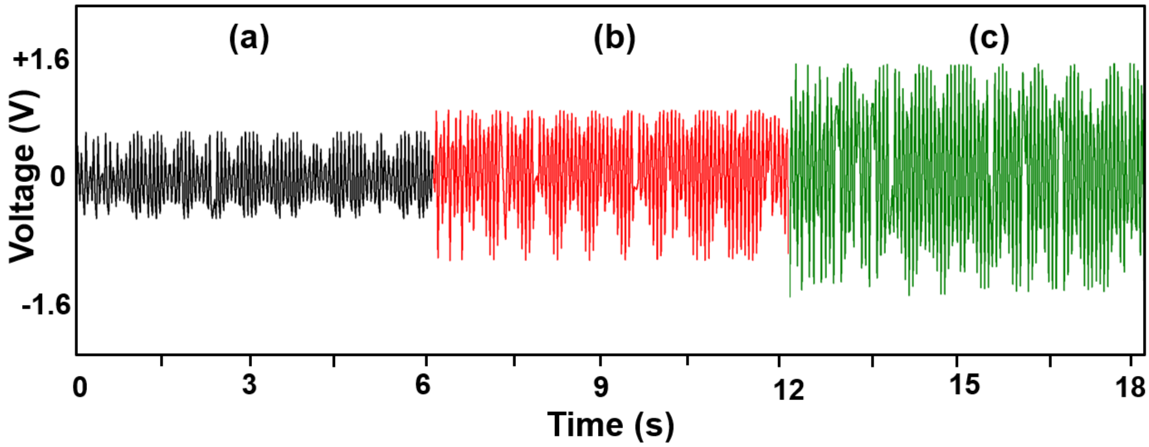

Fig. S8: The output voltage waveforms of the textured $BaTiO_3$-epoxy energy harvester with proof mass of (a) 10 g, (b) 17 g and (c) 24 g while driving voltage (15 V) and frequency (15 Hz) were kept constant.

Table S1. The change in generated output voltage of textured $BaTiO_3$-epoxy energy harvester with different optimization parameters of proof mass, signal voltage and frequency.

**S. No.**	**Proof mass (g)**	**Signal frequency (Hz)**	**Signal voltage (V)**	**Output V_{pp} (V)**
1	10	5	5	0.22
2	10	5	10	0.24
3	10	5	15	0.43
4	10	10	5	0.29
5	10	10	10	0.61
6	10	10	15	0.90
7	10	15	5	0.36
8	10	15	10	0.78
9	10	15	15	1.18
10	17	5	5	0.26
11	17	5	10	0.52
12	17	5	15	0.85
13	17	10	5	0.30
14	17	10	10	0.81
15	17	10	15	1.37
16	17	15	5	0.33
17	17	15	10	1.15
18	17	15	15	2.04
19	24	5	5	0.47
20	24	5	10	0.73
21	24	5	15	0.91
22	24	10	5	0.43
23	24	10	10	0.93
24	24	10	15	2.41
25	24	15	5	0.52
26	24	15	10	1.91
27	24	15	15	3.20
